# Nature and Age of Neighbours Matter: Interspecific Associations among Tree Species Exist and Vary across Life Stages in Tropical Forests

**DOI:** 10.1371/journal.pone.0141387

**Published:** 2015-11-18

**Authors:** Alicia Ledo

**Affiliations:** University of Aberdeen, Aberdeen, Scotland, United Kingdom; Institute of Botany, Chinese Academy of Sciences, CHINA

## Abstract

Detailed information about interspecific spatial associations among tropical tree species is scarce, and hence the ecological importance of those associations may have been underestimated. However, they can play a role in community assembly and species diversity maintenance. This study investigated the spatial dependence between pairs of species. First, the spatial associations (spatial attraction and spatial repulsion) that arose between species were examined. Second, different sizes of trees were considered in order to evaluate whether the spatial relationships between species are constant or vary during the lifetime of individuals. Third, the consistency of those spatial associations with the species-habitat associations found in previous studies was assessed. Two different tropical ecosystems were investigated: a montane cloud forest and a lowland moist forest. The results showed that spatial associations among species exist, and these vary among life stages and species. The rarity of negative spatial interactions suggested that exclusive competition was not common in the studied forests. On the other hand, positive interactions were common, and the results of this study strongly suggested that habitat associations were not the only cause of spatial attraction among species. If this is true, habitat associations and density dependence are not the only mechanisms that explain species distribution and diversity; other ecological interactions, such as facilitation among species, may also play a role. These spatial associations could be important in the assembly of tropical tree communities and forest succession, and should be taken into account in future studies.

## Introduction

The spatial pattern and arrangement of individuals of any species is fundamental in ecological theory, and numerous studies have focused on the spatial distribution of tropical tree species over the last few decades [1,2]. As a result of this, we now have more knowledge about how species are distributed within the forest, which has produced new insights into community assembly and the processes that contribute to small-scale structure [3]. The spatial distribution of tropical tree species is not random, but is aggregated [1] and differs among species [4]. The observed aggregation has been explained by two different underlying mechanisms, working at a reasonably small scale: dispersal limitations and habitat association. The main dispersal agent is directly responsible for the final observed pattern and cluster size of a species [4]. Furthermore, species with two main dispersal agents have a double cluster [2]. Habitat associations have also been found that act on large [5], medium [6], and small scales [7]. Another important mechanism acting on the spatial distribution of tropical tree species is negative density dependence [8]. This mechanism explains species distribution and diversity in tropical forests [9,10].

Most previous studies on species distribution in tropical forests have focused on the spatial distribution of each species separately, and the effect of heterospecifics has not received much attention [6, 11]. An important reason for this may be the difficulty in investigating a large number of species with a large number of interactions. Statistical models dealing with large amounts of data that contain complex interactions used to be slow [12]. Furthermore, one of the main biotic interactions acting on the distribution of trees and tree species is competition, the cornerstone interaction in density- and distance-dependence mechanisms [13, 14]. Under that theory, the interaction between individuals A and B is not direct competition, strictly speaking, but occurs via pests and pathogens that tree A passes to tree B. Nevertheless, the resulting pattern is the same: tree B will have poor survival in A’s vicinity. As researchers bear this well-accepted theory in mind, it has often led to them ignoring or diminishing the effects of other mechanisms, such as facilitation, which has hardly been considered in tropical studies [15, 16]. In fact, competition is a major factor reducing growth in neighbouring trees [13, 17], and may be one of the major biotic interactions acting on individual tree development. However, this does not indicate that competition (both above and below ground) is the only factor acting on species distribution and community assembly. Complex plant facilitation and mutualistic networks are key factors responsible for species distribution and coexistence in arid ecosystems [18], and the stress gradient hypothesis is based on this fact [19]. This is sufficient justification to explore whether facilitation also arises and plays a role in tropical ecosystem community assembly.

Positive and negative spatial associations (spatial attraction and repulsion, respectively) between species may be the result of various direct or indirect mechanisms and factors, either due to biotic/biotic or biotic/abiotic interactions. Spatial attraction between two species, in addition to direct facilitation or mutualism, may be due to a similar response to a common requirement, such as the amount of light [20], a particular disturbance [21] or soil nutrients [11]. It may also be due to a second facilitative agent, such as mycorrhizae, or due to indirect facilitation, such as where tree architecture creates an ecosystem with a determined structure required by another species [12]. Spatial repulsion, in addition to competition (a direct effect), can arise when one particular species prevents the development of another nearby species due to biochemical allelopathy (an indirect effect) [22]; it may reflect a divergent response to a given habitat [6, 7], or arise due to habitat filtering [6]. Moreover, we cannot assume that the factors that cause the observed spatial relationships among species are static. Recent research has revealed that tree-habitat associations change over different life stages [23], as do distance- and density-dependent mechanisms [3].

I hypothesized that spatial associations exist at short distances among tree species in species-rich forests, and those associations may change during an individual’s lifetime—that is, the associations and requirements of species are not constant over the lifespan, but are facultative and sequential. However, the importance of those spatial associations may be different among species belonging to different guilds. The spatial patterns of species can be studied to determine whether the observed patterns are consistent with underlying ecological mechanisms, discounting those that are not, and preserving as possible explanatory mechanisms those that agree with the patterns. Some spatial associations may be due to habitat preferences, but species-specific facilitative, mutualistic, and exclusively competitive processes also may arise. Confirming this hypothesis may be important to forest community ecology, and it shows that (1) there is likely to be a complex network among tropical tree species, as has been found in other ecosystems [18, 24]; (2) interspecific facilitation may also be important in determining species distribution, even though the effect of the facilitation process is frequently ignored [16]; (3) distinct succession and spatial arrangement of species exist in the forest [25]; (4) distinct species groups co-occur (and may coexist) in the forest, indicating that some species are spatially organized; and (5) it is further evident that the species found in tropical forests are not ecologically equivalent. Thus, these associations should be taken into account when predicting the species distribution within a forest.

In order to corroborate these hypotheses, a parallel analysis was performed in two tropical forests—a tropical montane cloud forest and a lowland moist forest. The observation of the expected pattern in both ecosystems would indicate that the spatial associations are not spurious, but generally exist in species-rich ecosystems. To obtain traceable and repeatable results, I studied the spatial relationships between pairs of species (hereafter called pairwise associations) instead of attempting to analyse them all together. The inclusion of all species together would probably mask important results and make interpretation extremely difficult. Pooling all the species together may partially obscure the effect of neighbourhood associations [17]. Similar pairwise techniques have been used to study plant networks [18, 24]. I studied the pairwise associations among species in both forests by first considering the individuals belonging to both species together, and then the pairwise associations of the individuals of each species at different life stages. Hence, I could draw the spatial associations with and without considering variations at different plant life stages.

Additionally, I examined whether pairwise associations were more common between species with similar life forms and shade tolerances. The debate on functional characteristics will not be addressed in this study because it is beyond the scope of the current research. Nevertheless, I bring this information up here because it offers a broader view on forest organization, and it could be more fully examined in future studies.

Cloud forests are among the least understood and least studied ecosystems in the world [26], and hence, the ecological mechanisms that operate in these forests remain relatively unknown. Moreover, cloud forests are one of the most seriously threatened ecosystems [26]; hence, studies on their functioning and maintenance are of particular importance.

## Materials and Methods

### Study sites and vegetation sampling

The first study site was in the Peruvian cloud forest ‘Bosque de Neblina de Cuyas’ (BNC), 4°35ʹS, 79°42ʹW. The elevations range from 2359 to 3012 m above sea level and are characterised by irregular surfaces and steep slopes. Accordingly, the forest stand is unevenly aged, and has a high rate of biodiversity and endemism [4]. This forest is one of the last original cloud forests on the Pacific slope of the Andes in Northern Peru. Although the zone has been identified as an Important Bird Area (IBA005), the forest has no other protection or conservation. This forest belongs to the local communities inhabiting the areas, named ‘Comunidad Campesina de Cuyas Cuchayo Ambasal’, who have declared it a private conservation area. I have a verbal agreement with them that allows me to work there; they have no written records yet. Nevertheless, the forest faces a serious threat because it is probable that mining activity will begin in the region, as it has in many areas of the Andes. I conducted an inventory in 2008 by establishing three different 1-ha square plots located in the old-growth stand. Physiography and environmental conditions were relatively homogenous across all three plots [7]. As the plots were similar, the patterns identified for each species in the different plots can be considered replicates of the same ecological processes. In each plot, all the free-standing woody plants taller than 1.3 m were mapped and the diameter at breast height (DBH), height, and species were recorded. A list of the characters for the species found, including their life form and shade tolerance, is provided in Supplementary material S1 Table, additional information on the plots and the species has been published previously [4]. BNC census data are given in Supplementary material S1 Data and more data are freely available from http://www.alicialedo.com. The BCI data is available upon request at http://ctfs.arnarb.harvard.edu/webatlas/datasets/bci/

The second study site was Barro Colorado Island (BCI), a seasonally dry tropical moist forest, 9°10′N, 79°51′W, in Panama [27, 28]. A 50-ha permanent plot was established in this well documented forest in 1982. Every 5 years, all free-standing trees in the plot ≥ 1 cm in diameter are re-measured in the plot. BCI is managed by the Smithsonian Tropical Research Institute (STRI), and it forms part of the Barro Colorado Nature Monument (BCNM). I used data from the 6th census on BCI, which was conducted in 2005. Due to the large size of the plot, diverse areas in relation to the topography can be observed, and six different habitats have been described within the plot: low plateau, high plateau, slope, swamp, streamside, and young forest [6].

Both study areas are neotropical forests, although there are some differences with regard to the ecosystems, environmental conditions, inventory, and plot size. BNC is a montane cloud forest, whereas BCI is a lowland seasonal forest. Therefore, BNC experiences a colder climate, but is less affected by seasonal changes in humidity than the BCI forest [7]. Nevertheless, the overall spatial pattern of the tree species is similar at both sites: the tree species in both BNC [4] and BCI [1] show clustered distributions, which are typical for most of the tropical forests analysed to date [2]. Hence, the ecological processes producing the spatial pattern of the different species, dispersal limitations [1], and niche preferences or habitat filtering [6] operate in both studied forests. However, as the plot size differed considerably between the sites, so did the heterogeneity within the studied areas. BNC plots were more homogeneous in terms of environmental variation than the BCI plots because a smaller area was examined, because of which different habitat types could not be differentiated in BNC as they have been in BCI [6]. Accordingly, BNC plots can be considered a single habitat under a habitat-partitioning definition that is similar to the BCI habitat definition. Hence, to avoid the effect of species-habitat associations in the BCI plot and, thus, be able to compare the plots in terms of the environmental variability of the two study areas, spatial analyses for pairs of species characteristics from each of the defined habitats were performed. Thus, the BCI species considered (S2 Table) were those that are associated with a particular habitat (p-value < 0.01), following the results of Smith et al [7]. A list of the characteristics for the species found, including life form and shade tolerance, is also provided in S2 Table. However, it is important to note that spatial variability and microhabitat associations also exist within BNC [7] and BCI habitats [29]. While the methods used in this study should enable the study plots to be compared in terms of environmental heterogeneity, the microhabitat effect may not have been totally removed. Indeed, there may be more environmental variation in BNC, because it is a mountain forest with a marked slope, which may lead to a micro-environmental gradient with elevation, absent in BCI. This suggests that BNC trees are under greater environmental stress.

### Statistical analysis

A multivariate point pattern analysis approach was used to study the spatial association between species [30, 31]. The point pattern analysis technique describes the second-moment properties of the spatial pattern of a point layer as a function of the inter-point distance as the distance of observation increases. Hence, this approach allows the detection of the observed spatial pattern of a point layer at different scales. Currently, point pattern techniques are widely used in ecology to analyse the spatial structure of the elements that make up ecosystems.

To assess the pairwise associations between species when considering the individuals of both species together, this study employed the bivariate function [32], which is used to analyse the spatial association (attraction, repulsion, or spatial independence) between two types of points, which in this analysis were both woody plant species.

$${\hat{L}}_{rs}(d)=\sqrt{\frac{1}{(\lambda_{r}+\lambda_{s})(n_{r}+n_{s})\pi }}\left(\sum_{i=1}^{n_{r}}\sum_{j=1}^{n_{s}}\frac{\omega_{ij}(d)}{n_{r}}+\sum_{i=1}^{n_{s}}\sum_{j=1}^{n_{r}}\frac{\omega_{ij}(d)}{n_{s}}\right)$$ (1)

where *d* is the distance lag of the analysis, *n*_*r*_ and *n*_*s*_ are the number of trees in each class, λr=nrA and λs=nsA, respectively (*A* being the area of the plot), and *ω*_*ιj*_ is the second-order moment measure, $\omega_{ij}(d)=\left\{ \begin{array}{c} 1\;if\;d_{ij}\;\leq d\\ 0\;if\;d_{ij}\;>d \end{array}\right.$, which incorporates the boundary-effect correction proposed by Ripley (1979).

**Fig 1:**
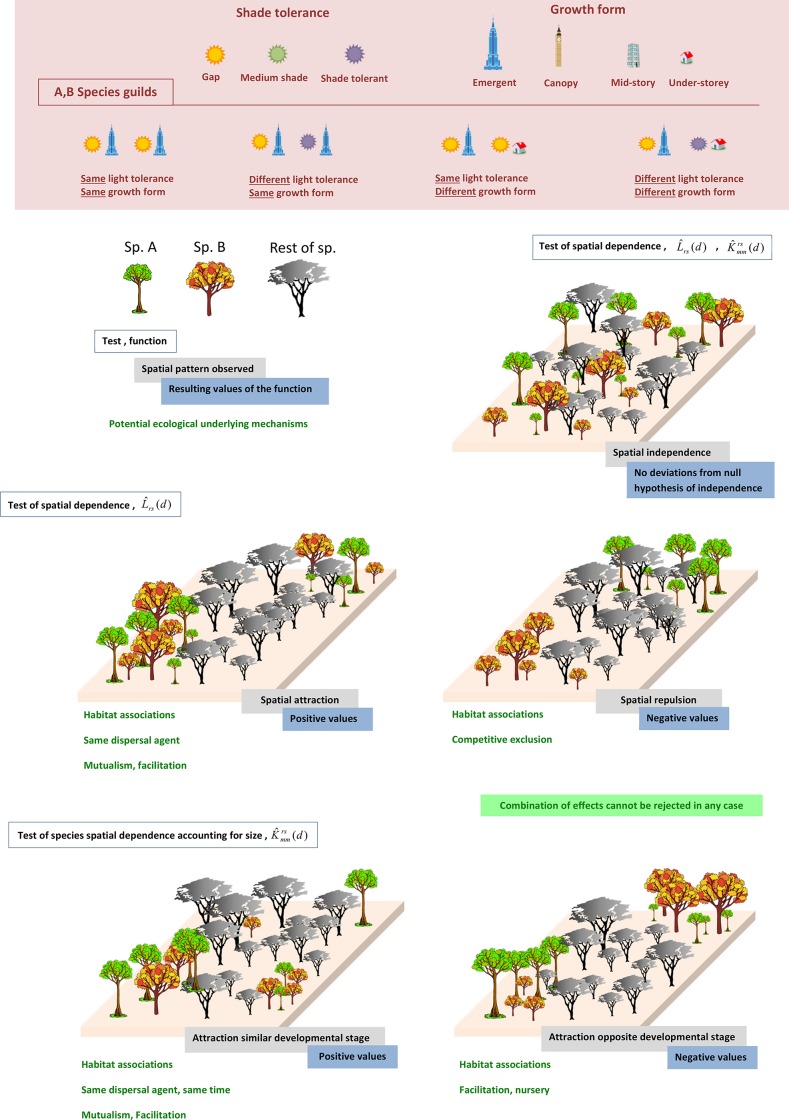
Conceptual framework detailing the potential spatial patterns between species that can be detected with the point pattern functions used in this study. In every of the 5 different cases, the intertype function used to detect the spatial dependence is in a white box, the observed spatial dependence and the values in a grey box, the function that support that result is in a blue box. The potential underlying mechanisms that caused the observed pattern are indicated in green characters. Only the species comprising the pair of target species were represented in this scheme, named species A and B, while the other species were blind. Species A and B may be any pair of species, different in each case. Rosy upper box: light tolerance and growth form classification of the analysed species. The four different cases of inter cross guilds for species A and B are represented below.

The toroidal shift null model [33] was constructed to test the spatial independence between the species. In the toroidal shift model simulation, the position of the points of one class is left unchanged, while all the points of the other class are shifted by the same random vector, and the area is considered a torus. Deviations from the model would imply spatial attraction (if the empirical function is above the 95% quantile) or spatial repulsion (if the empirical function is below the 95% quantile) between the species included in the analysis. A schematic visualization of those processes can be found in Fig 1.

To assess the pairwise associations between species when considering their different developmental ages, the intertype mark correlation function, ${\hat{K}}_{mm}^{rs}(d)$ was used, which we proposed and tested previously [34] and is used for the first time in this analysis. This function assesses the expected covariance of the marks between pairs of points belonging to two different tree types within a given distance, which means that it is a pairwise and mark correlation function. The trunk diameter was considered as a surrogate of an individual’s developmental stage in this analysis. In tropical forests, seedling growth can be supressed for years until a high light opportunity presents itself [35], so smaller trees can be decades old, but, as they are waiting for an opportunity to grow rapidly, I considered them to be individuals in the sapling developmental stage. Besides, although the trunk diameter is not exactly correlated with age, it is reasonable to consider that larger trees of a given species (50–70 cm DBH) are older than the smaller conspecific trees (1–5 cm DBH). Hence, I introduced the diameters as marks, being a continuous variable, and the two types of points as two different species. It should be pointed out that, as observed in the ${\hat{K}}_{mm}^{rs}(d)$ function, the range of the two marks is normalised in the function, allowing the comparison of two species with different diameter ranges. Because I had considered the diameter as a measure of tree size and, thus, a surrogate of developmental stage, the results of the function would allow the identification of the spatial relationships between two species when considering younger individuals of one species versus older individuals of the other, and vice versa.

$${\hat{K}}_{mm}^{rs}(d)=\frac{\left(\sum_{i=1}^{n_{r}}\sum_{j=1}^{n_{s}}\omega_{ij}(d)\frac{\left(m_{i}-\bar{m_{r}}\right)}{s_{r}}\frac{\left(m_{j}-\bar{m_{s}}\right)}{s_{s}}+\sum_{i=1}^{n_{s}}\sum_{j=1}^{n_{r}}\omega_{ji}(d)\frac{\left(m_{i}-\bar{m_{r}}\right)}{s_{r}}\frac{\left(m_{j}-\bar{m_{s}}\right)}{s_{s}}\right)}{\sum_{i=1}^{n_{r}}\sum_{j=1}^{n_{s}}\omega_{ij}(d)+\sum_{i=1}^{n_{s}}\sum_{j=1}^{n_{r}}\omega_{ji}(d)}$$ (2)

where *m*_*i*_ and *m*_*j*_ are the values of the mark at the points *i* and *j*, respectively, and *s*_*r*_ and *s*_*s*_ are the standard deviation of the mark of the points considered in class *r* and class *s*.

The random marking null model [34] was used to test the spatial independence of the diameters. The random marking null model tests independence in the mark distribution when the spatial pattern of both point classes is fixed and the mark is randomised. If the trees of two species with similar normalised values for trunk diameter coexist at a certain scale, the larger (or smaller) individuals of one species and the larger (or smaller) individuals of the other will appear to be spatially related when the empirical function is above the 95% quantile. In contrast, the function will be below the 95% quantile bounds if species having opposite diameter values appear spatially together, that is larger individuals of one species occur with smaller individuals of the other species. If the diameters of the two species are independently distributed, the empirical function would be within the quantile bounds, regardless of the spatial pattern. A schematic visualization of those processes can be found in Fig 1.

For BNC only, I calculated the overall ${\hat{L}}_{rs}(d)$ and ${\hat{K}}_{mm}^{rs}(d)$ functions by standardising and replicating the Eq 1 and Eq 2 functions obtained in the tree plots. I used a standardisation method [36]. The standardized function (${\hat{L}}_{rs}(d)$ for Eq 1 and ${\hat{K}}_{mm}^{rs}(d)$ for Eq 2) for each *r* replicate was standardized by the transformation: ${\hat{F}}_{r}^{st}(d)=a(d)+b(d){\hat{F}}_{r}(d)$; where ${\hat{F}}_{r}^{st}(d)$ is the target function (either Eqs 1 or 2), $a(d)=1-b(d)N_{r}^{sup}(d)$ and $b(d)=\frac{2}{N_{r}^{sup}(d)-N_{r}^{inf}(d)}$, with $N_{r}^{sup}(d)$ and $N_{r}^{inf}(d)$ representing the upper and lower 95% quantile bounds, respectively, of the null model. I translated the 95% upper and lower quantile bounds ($N_{r}^{sup}(d)$ and $N_{r}^{inf}(d)$ of the null model to between –1 and 1 for each distance *d* to make them comparable. Then, the overall standardized function was calculated for the three replicates:

$${\hat{F}}^{st}(d)=\frac{{\hat{F}}_{r}^{st}(d)}{3}$$ (3)

Type II errors (false positives) were minimised in the BNC approach because the results from three different spatially separated plots were used. Therefore, the casual associations in one plot were removed, and only coincident results were considered, making the results more robust.

In the spatial analysis, I included only species with more than 60 individuals in each of BNC plots and in the BCI plot. In BNC forest, a few species had fewer than 60 individuals in only one of the three plots. When this occurred, the results for this species in that plot were not included in the analysis and only the two plots with a sufficient number of individuals were included in the replicate analysis (Eq 3). Although this approach excluded rare species, these are seldom included in spatial pattern analysis, and ignoring them does not induce a notable bias [2]. Hence, 20 species were included in the BNC analysis, comprising 40% of the species found and 93% of the total woody plant individuals in the measured plots (S1 Table). Twenty-five species (associated with a habitat and with more than 60 individuals) were analysed in the BCI plot (S2 Table).

A total of 399 simulations were performed to build the quantile boundaries of the null models, and I designated 95% of the quantile boundaries of the null model as the area corresponding to an acceptance of the null hypothesis. Moreover, I calculated the quasi p-values of the analysis using a previously described method [37] and considered a significance level of 0.01 to mean acceptance. The distance considered in this analysis was 50 m. These values corresponded to half the length of the shorter dimension of the BNC plots, as previously recommended [30].

Both the ${\hat{L}}_{rs}(d)$ and ${\hat{K}}_{mm}^{rs}(d)$ functions revealed all the scales at which deviations from the null hypothesis occur, which are considered to be an interval. Nevertheless, to synthesise the results, I considered a single distance at which a spatial interaction arose, which was also the point at which the spatial association was more significant, i.e. the point at which the difference between the empirical function and the limit band of acceptance (the upper and lower quartile, respectively) was greater, as weighed by the distance interval area of the null hypothesis acceptance.

As a large number of tests were carried out, 5% of the associations were expected to be false positives (type I errors), which equates to four associations in each of the two analyses (with and without including trunk diameter) in BNC and six in each of the two analysis for BCI, based on the number of statistical tests (20 and 25 species, respectively) that were conducted.

After the above analyses and based on the results of the pairwise associations, I examined whether pairwise associations were more common between species with similar life forms and shade tolerance. This may also indicate species arrangement and forest organization. The life forms and shade tolerance of species from BNC are in [4] and S1 Table. The life forms of species from BCI are described in [23] and presented in S2 Table. Each functional trait has been represented by a different symbol in Fig 1, which is used throughout the manuscript to represent that particular functional trait.

### Pairwise associations and habitat associations

In order to check whether the found pairwise associations are due to a pairwise concordance in habitat associations, I compared the results obtained from examining the pairwise associations with the microhabitat associations found in [7] for BNC. If the spatially associated species were also associated to a similar habitat, the spatial association is probably due to a positive response to similar habitat conditions. If the species are not associated with the same microhabitats, then it can be assumed that the underlying process that causes the pairwise associations may be due to a different process. An analogous analysis could not be done in BCI. Species microhabitat association results do not exist and were not within the scope of this study.

## Results

### Spatial associations between species in a tropical cloud forest

**Fig 2:**
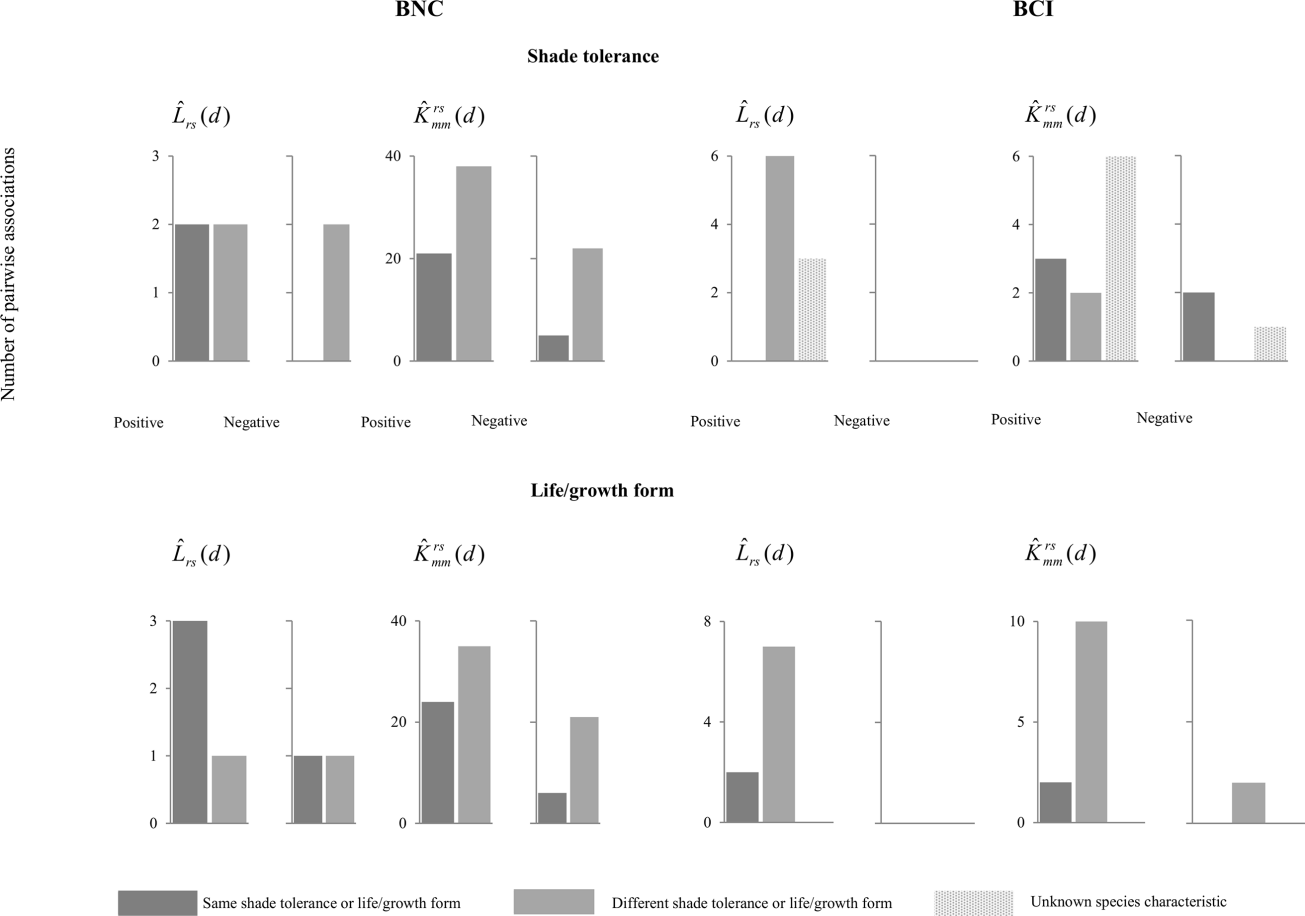
Number of pairwise association between species with similar (dark grey) or different (light grey) dispersal modes, shade tolerance and life-forms found using the ${\hat{L}}_{rs}(d)$ and ${\hat{K}}_{mm}^{rs}(d)$ analyses for positive and negative associations in BNC. In ${\hat{L}}_{rs}(d)$, positive and negative associations indicate spatial attraction and repulsion, respectively. In the ${\hat{K}}_{mm}^{rs}(d)$ analysis, positive values indicate attraction among species at similar ages, and negative values indicate attraction among species at opposite ages.

Fourteen species, out of the 20 species included in the analysis, showed some spatial association with other species, according to the ${\hat{L}}_{rs}(d)$ analysis (Table 1). Twelve were positive and two were negative. The interaction distances ranged from 0.5 to 46 m, but most of them were at short distances of up to 10 m (Table 1, lower left). They represent 7% of the potential associations (a hypothetical scenario where all the species have spatial associations). For those pairs of species that exhibited spatial repulsion, it was noted primarily at a small scale, with maximum distances for the spatial association reaching 1 m. Most of the pairs showing spatial associations were between species that had similar life forms (Fig 2, Table 1) and, hence, coexisted in the same vertical layer of the stand. For example, *Parathesis* Ms and *Eugenia* Ms showed attraction at a small scale, and these species are the most frequent under-canopy shade-tolerant species in the lower layer of the forest. *Persea* Ms and *Morus insignis* are both canopy species, and also appeared spatially associated at a small scale (Table 1). Species with different shade tolerances generally showed spatial repulsion (Fig 2, Table 1).

**Table 1:** Matrix of results from BNC: (i) the lower and left half of the matrix indicates the distance (metres) at which a spatial association was found using the ${\hat{L}}_{rs}(d)$ analysis. A positive value indicates attraction, and a negative value indicates repulsion: (ii) the upper and right half of the matrix indicates the distance at which a spatial association was found using the ${\hat{K}}_{mm}^{rs}(d)$ analysis. If a positive value indicates attraction among species at similar life-stages, then a negative value indicates attraction among species at opposite life-stages. In every case, the distances correspond to the point at which the spatial association is more important. The numbers in italics are the number of spatial associations that each species exhibited, corresponding to the results plotted in that half of the matrix. The cells marked in bold indicate that that pair of species showed similar association to a habitat (Ledo et al 2013), if the relation between species is positive or showed attraction and repulsion to a habitat if the relation is negative. Ms refers to Morphospecies, from Ledo et al. 2012.

BNC		*Cestrum auriculatum*	*Critoniopsis sevillana*	*Delostoma integrifolium*	*Drimys* Ms	*Eugenia* Ms	*Iochroma squamosum*	*Lycianthes inaequilatera*	*Meliosma* Ms2	*Miconia media*	*Miconia denticulata*	*Miconia firma*	*Morus insignis*	*Oreopanax raimondii*	*Parathesis* Ms	*Persea* Ms	*Piper elongatum*	*Ruagea glabra*	*Solanum* Ms1	*Solanum* Ms2	*Tournefortia* Ms2
		*8*	*5*	*6*	*6*	*6*	*13*	*12*	*3*	*11*	*3*	*14*	*7*	*9*	*14*	*4*	*16*	*7*	*14*	*6*	*8*
*Cestrum auriculatum*	*1*							-19	2		9.6		39		27	39		7.1		0.5	
*Critoniopsis sevillana*	*1*						35	-7.1		-2.5							-13		-10		
*Delostoma integrifolium*	*0*						6.1						-40	26			37	**19**	11		
*Drimys* Ms	*0*							6.1						13	6.1		1		0.5		-4
*Eugenia* Ms	*1*						-0.5	-10	-0.5			26			28		19				
*Iochroma squamosum*	*1*							-21		-36		5.6	-11	-2.5	-25		-21		**-50**		37
*Lycianthes inaequilatera*	*0*									-2.5		-24		38	25				4.5	18	-32
*Meliosma* Ms2	*0*														-1		-1.5				
*Miconia media*	*1*										16	**7.1**			**12**	**0.5**	4.5	48	**16**		
*Miconia denticulata*	*1*		46									23						-43			
*Miconia firma*	*4*												-0.5		13	-31	13	-43	8.5	24	8.6
*Morus insignis*	*2*											1			**22**	**8.6**				**0.5**	15
*Oreopanax raimondii*	*2*														0.5		10	18	32		
*Parathesis* Ms	*2*					3					40						4		37	9.3	18
*Persea* Ms	*1*						-0.5										-6.1				
*Piper elongatum*	*3*									8.6		**4.5**		**3.5**				32	**7.6**	**5.1**	9.6
*Ruagea glabra*	*4*	**-0.5**										**10**	2	**16**					11		
*Solanum* Ms1	*0*																			**6.1**	0.5
*Solanum* Ms2	*1*											4									
*Tournefortia* Ms2	*0*																				

The number of spatial associations found when diameter size was accounted for in the analysis was notably larger than in the previous analyses, as derived from the ${\hat{K}}_{mm}^{rs}(d)$ results (Table 1). In this second case, 86 interactions among the pairs of species were found (59 positive and 27 negative), with distances ranging from 0.5 to 47 m (Table 1, upper right). In this case, positive associations meant spatial attraction between species at a similar developmental stage and negative associations meant spatial attraction between species at different developmental stages. The number of observed associations represented 45% of the potential associations. In addition, associations were found between species in similar vertical layers and also between species with different life forms (Fig 2, Table 1). The under-canopy species had a greater number of associations than the other life forms (Table 1, S1 Table). The species with the greatest numbers of associations were *Solanum* Ms1 and *Piper elongatum*, both pioneer species (S1 Table). Once again, positive associations, in this case, meaning spatial attraction among species of similar ages, appeared to be more common between species with different shade tolerances and life forms (Fig 2, Table 1).

### Spatial associations between species in a tropical lowland moist forest

Fourteen species, out of the 25 species included in the analysis, showed some spatial association with other species, according to the ${\hat{L}}_{rs}(d)$ analysis (Table 2). The total number of spatial interactions in this second forest was nine and all of them were positive, with distances ranging from 7 to 40 m (Table 2). That represents 15% of the potential associations. No associations were found in the selected species in the high plateau and stream habitats. However, this may be due to the lower number of species included in this analysis, and hence we cannot make any ecological assumptions based on this result. In this analysis, it was observed that most of the spatial associations were between different growth form species, such as *Cordia bicolor* (midstorey) and *Coussarea curvigemmia* (understorey), or *Ocotea oblonga* (canopy) and *Xylopia macrantha* (midstorey). However, no clear pattern was observed for species shade tolerance (Table 2), which was mainly due to the lack of information on this characteristic (Fig 2, Table 2).

**Table 2:** Results of the ${\hat{L}}_{rs}(d)$ and ${\hat{K}}_{mm}^{rs}(d)$ analyses for the species considered in each habitat in the BCI plot. *S* indicates the number of species analysed in the habitat, m indicates the distance (meters) at which a spatial association was found. In the ${\hat{L}}_{rs}(d)$ analysis, if positive means attraction, then negative means repulsion. In the ${\hat{K}}_{mm}^{rs}(d)$ analysis, if positive means attraction among species at similar life-stages, then negative means attraction among species at opposite life-stages.

	${\hat{L}}_{rs}(d)$		${\hat{K}}_{mm}^{rs}(d)$	
Habitat	Species	m	Species	m
	*Cordia bicolor—Coussarea curvigemmia*	10 to 40	*Cordia bicolor—Mouriri myrtilloides*	30
	*Coussarea curvigemmia—Ouratea lucens*	8 to 30	*Cordia bicolor—Ouratea lucens*	-10
**Low plateau**	*Ouratea lucens—Tetragastris panamensis*	5 to 40	*Coussarea curvigemmia—Ouratea lucens*	35
*S* = 7	*Protium panamense—Simarouba amara*	7	*Coussarea curvigemmia—Tetragastris panamensis*	19
			*Mouriri myrtilloides—Protium panamense*	8
			*Ouratea lucens—Tetragastris panamensis*	15
			*Protium panamense—Simarouba amara*	7
	*Drypetes standleyi—Hirtella triandra*	30	*Chrysophyllum argenteum—Hirtella triandra*	17
**Slope**	*Ocotea oblonga—Xylopia macrantha*	18	*Drypetes standleyi—Hirtella triandra*	20
*S* = 8	*Ocotea oblonga—Ocotea whitei*	20	*Hirtella triandra—Trophis caucana*	-5
			*Hirtella triandra—Xylopia macrantha*	-20
**Swamp**	*Alibertia edulis—Sychotria graciliflora*	12	*Alibertia edulis—Cassipourea elliptica*	-5
*S* = 4	*Alibertia edulis—Tabernaemontana arborea*	23	*Cassipourea elliptica—Psychotria graciliflora*	7
			*Cassipourea elliptica—Tabernaemontana arborea*	5
**High plateau** *S* = 3	*No significant associations*		*No significant associations*	
**Stream** *S* = 3	*No significant associations*		*No significant associations*	

Once again, the number of spatial associations found when diameter size was accounted for in the analysis was larger than in the previous analyses, although the difference was not as evident as in the cloud forest. Fourteen interactions among pairs of species were found by the ${\hat{K}}_{mm}^{rs}(d)$ analysis (10 positive and 4 negative), with distances ranging from 5 to 35 m (Table 2). That represents 23% of the potential associations. Most of the pairwise associations were positive, but some were negative, as the case of *Alibertia edulis* and *Cassipourea elliptica* (Table 2). Most pairs of associated species had different growth forms (Fig 2, Table 2), such as *Coussarea curvigina* (understorey) and *Tetragilis panamensis* (canopy). However, pairwise associations were also found for species with the same growth form, such as *Protium panamense* and *Simarouba amara*, both of which are canopy species (Table 2).

### Pairwise associations, light tolerance, and growth form

The undercanopy and shade species showed a greater number of associations with other species than the canopy and gap species in both forests (Tables 1 and 2). In the montane BNC, the percentage of spatial associations regardless of pairs of species belonging to a similar shade-tolerance group was the same as the percentage of pairs of species belonging to a different group, but spatial repulsion occurred only for species with different growth forms. In the lowland BCI forest, the pattern for positive spatial associations was the opposite: species with positive associations belonged to different groups. As for growth form, species had positive spatial associations with species mainly belonging to the same group in BNC, but with species from different groups in BCI (Fig 2). When size was included in the analysis, pairs of associated species mainly belonged to different guilds for both light tolerance and growth in BNC. In BCI, this pattern was observed only for growth form (Fig 2).

### Pairwise associations and habitat associations in BNC

Five out of the 14 spatial interactions derived from the ${\hat{L}}_{rs}(d)$ analysis (36% of the total spatial associations) were also found in the habitat association analysis (Table 1). That means, for five of the 14 found spatial interactions the pairs of species either showed spatial attraction and were associated with a particular microhabitat or showed spatial repulsion where one species was associated to a habitat whereas the other avoided that habitat.

When plant size was considered in the ${\hat{K}}_{mm}^{rs}(d)$ analysis, 12 out of 86 interactions (14% of the total spatial associations) followed the aforementioned pattern, in which both had a positive association and exhibited either a positive or a negative association with a particular microhabitat (Table 1).

Spatial associations that could be due to microhabitat associations, because they were also consistent with this pattern, were found mainly between three species: *Miconia media*, *Morus insignis*, and *Piper elongatum*. Both *M*. *media* and *P*. *elongatum* species are pioneer, undercanopy species (S1 Table). The distribution of these species may be related to gaps. *M*. *insignis* is a species frequently associated with streams or wetter habitats, so it may be more sensitive to and dependent on particular habitat types [7].

## Discussion

The results confirmed the proposed hypothesis: spatial associations between species in species-rich forests exist and they do change as individuals age. Apart from the conspecific spatial interactions (density dependence, dispersal limitations), heterospecific spatial associations between different species also exist, and play a role in species distribution within the forest. Even though competition among trees is always present in dense forest, most of the spatial interactions were positive, contradicting the pattern that should arise from competition. Further, most of those positive associations were inconsistent with habitat associations under the tree habitat definition that had been tested (Table 1, [7]), indicating that another mechanism may create this observed pattern. Among those other factors, a mechanism that results in spatial association is facilitation, either direct or indirect. Hence, our spatial pattern finding agree with the idea that facilitation may play a role in species allocation and distribution in species-rich forests, and seems to be more important during the early growth stages. Some species appear to form specific species-groups and are very often in cohorts. Hence, there are interspecific associations acting on species distribution, which may also have important effects on community assembly and species diversity maintenance.

### Spatial pairwise species associations

A pattern of species associations that is inconsistent with a random distribution and supports species spatial associations, both positive (spatial attraction) and negative (spatial repulsion or desegregation), has been clearly shown in both study areas (Tables 1 and 2). The pairs of species that were positively spatially associated increased notably in both forest types when tree size was considered (Tables 1 and 2). This indicates that the spatial preferences and associations between species are not constant throughout the life of trees, and nor are species-habitat associations [23] and distance- and density-dependence mechanisms [3]. The positive spatial associations found may arise from a few underlying mechanisms. One of the most explored and recognized mechanisms is habitat associations [6]. We expect two species to be spatially related if they appear to prefer the same habitat. Indeed, in the current analyses, few of the observed positive pairwise spatial associations may be due to habitat associations, whether or not the tree size of the individuals is taken into account. Nevertheless, those associations were found for pioneer species in this particular case; therefore, this may be due either to a dependence on specific canopy disturbance conditions, or a response to a determinate amount of light [20, 37]. Nevertheless, the percentage of species associated with a habitat may be larger if we consider the ‘habitat’ definition in a wider context, i. e. including a particular mycorrhizal community, or microbiota existing in the soil. As that characteristic was not considered in the former habitat partitioning, we cannot support or discard it because no data are available; therefore, habitat associations may be more numerous than the ones we found. However, we cannot rule out another effect: that some species are in the same habitat due to a spatial pairwise positive association (facilitation or mutualism), which is not due to habitat associations. Further, a third situation could also be possible: a few species may be close together due to a combination of both effects—habitat associations and facilitative interactions—acting simultaneously.

As the effect of a similar response to a habitat cannot be assumed to be the sole mechanism underlying positive associations, we cannot discard the possibility that facilitation (both direct and indirect) between species may also exist in the forest. Two different cases can be discerned here: most of the pairwise spatial associations were found when the life stages of the species were similar, which indicated the existence of species-specific cohort groups. Alternatively, associations between mature individuals of one species and younger individuals of the other species may indicate that a nursery could exist in the forest. Those associations were found mainly among species with different shade tolerances and growth forms (Table 1, Fig 2). Hence, the observed patterns suggest that some undercanopy species appear mainly close to a specific canopy species, and vice versa. This is interesting because these species associations indicate that some successional species-directed group of species appears in the forest. This agrees with the successional niche hypothesis [25]. However, nursery effects were not observed in the studied BCI species. BNC, a montane cloud forest, is found on steep slopes, which are exposed to more variable temperature conditions, lower light levels, and high wind speeds in the dry season. The environmental conditions are not as benign as in lowland tropical forests. This suggests that BNC trees are under greater environmental stress. The postulated stress hypothesis [15, 19] states that facilitative plant-plant interactions are more important and more intense in high-stress environments. Nevertheless, despite finding a potential nursery effect, we cannot discard that this may occur only at an early stage but end with the small tree being suppressed by the larger tree in the future [37]. If so, we are observing facilitation at a particular point but not throughout the life stages. This, however, agrees with the idea that those spatial associations are not constant throughout life.

Another possible explanation (and perhaps the simplest) for the positive spatial interaction in the forest is that both species were dispersed into the space by different dispersers, at roughly similar times, such as when a gap was formed [38]. Alternately, the same dispersal agent, such as a particular bird or mammal, lives in a preferential area or habitat in the forest and so disperses both species in the same areas. Hence, the dispersal mechanism could also explain some of the observed pattern.

Negative pairwise spatial associations, which reflect repulsion between species, were never found in BCI (Table 2) and seldom in BNC (Table 1). Furthermore, there were very short distances (less than 1 m) between these associations, which is probably due to the physical impediment of trees growing close to each other [4]. This suggests that competitive exclusion between species [39] was not observed in the studied forests, which explains why tropical forests contain large numbers of species [8]. It also indicates that pairwise direct allelopathy [22] is not commonly present among tropical trees. However, it should be pointed out that repulsion among trees does exist in tropical forests due to competition [13, 14], but this competition for space, light, and nutrients may be on a small scale and be species-generalist, rather than species-specific. Direct experiments are needed to determine the mechanisms and causes responsible for the observed pattern of spatial associations.

### Implications for community assembly

It is well known and acknowledged that both niche preferences and distance-dependence mechanisms are involved in species’ spatial patterns and community assembly [8, 40]. The importance of these processes varies among species: some species are more sensitive to habitat conditions [7, 40], others to density dependence [9, 10], disturbance dependence [28, 41], or the characteristics of their neighbours, as the results of this study showed. This indicated that not all species are sensitive to or affected by a particular factor to the same degree, but differ in their responses to those ecological mechanisms. Consequently, different species appear to be sensitive in various degrees to different mechanisms; for species with stronger habitat preferences, habitat association may be the main mechanism determining the final distribution of the species, and rare species are more strongly affected by density dependence than common species [9, 10]. Specific spatial associations with a particular paired species may also play a notable role in the distribution and development of some species, as shown in this study. Spatial associations appear frequently between species with different functional traits (Fig 2), indicating that functional traits may be important and are predictors of forest organization, agreeing with the previously suggested idea [17]. Furthermore, the aforementioned mechanisms not only act together, but in a hierarchical way. A species may first need suitable environmental and stand conditions, which can either be a determinate forest structure or a response to a disturbance. Under those conditions, density dependence mechanisms would appear. Moreover, if a companion species is present, it may facilitate the development of the target species at that point, which may otherwise fail.

Facilitation among species has increased in importance in evolutionary theory [24]. Possibly, it is time to presume, or at least consider, the idea of a network of facilitative trees and incorporate it into the ecology of tropical tree coexistence. Most of the analysed species displayed more than one pairwise association (Tables 1 and 2). This finding suggests the existence of a mutualist network among species in tropical community forests, as described for arid ecosystems [18]. It is also necessary to consider the developmental stage of the individuals in community assembly. This study shows that age-independent analysis of species associations, the most common analyses done to date, underestimates interspecific plant spatial associations.

## Supporting Information

S1 Data BNC plots, listing all the measured trees indicating species, coordinates, DBH and tree height. (ZIP)

S1 Table Botanical list of the species observed, detailing the life form, shade tolerance and number of averaged individuals in the three plots, from Ledo *et al*. 2012. Ms refers to Morphospecies.
(DOCX)

S2 Table Species analysed in each habitat in the BCI plot, detailing the habitat at which are associated, life form and shade tolerance, from Comita et al 2007, and number of individuals. (DOCX)

## References

[1] Condit R, Ashton PS, Baker P, Bunyavejchewin S, Gunatilleke S, Gunatilleke S, et al 2000 Spatial patterns in the distribution of tropical tree species.—Science 288: 1414–1418. 10827950 10.1126/science.288.5470.1414

[2] Wiegand T, Gunatilleke S, Gunatilleke N, Okuda T. 2007 Analyzing the spatial structure of a Sri Lankan tree species with multiple scales of clustering.—Ecology 88: 3088–3092. 18229843 10.1890/06-1350.1

[3] Hu YH, Sha LQ, Blanchet FG, Zhang JL, Tang Y, Lan GY, et al 2012 Dominant species and dispersal limitation regulate tree species distributions in a 20-ha plot in Xishuangbanna, southwest China.—Oikos 121: 952–960.

[4] Ledo A, Montes F, Condés S. 2012 Different spatial organisation strategies of woody plant species in a montane cloud forest.—Acta Oecol. 38: 49–57.

[5] Gentry AH. 1988 Changes in plant community diversity and florsitifc composition on environmental and geographical gradients.—Ann. Missouri Bot. Gard. 75: 1–34.

[6] Harms KE, Condit R, Hubbell SP, Foster RB. 2001 Habitat association of trees and shrubs in a 50-ha neotropical forest plot.—J. Ecol. 89: 947–959.

[7] Ledo A, Burslem DFRP, Condés S, Montes F. 2013 Micro-scale habitat associations of woody plants in a neotropical cloud forest.—J. Veg. Sci. 24: 1086–1097.

[8] Wright JS. 2002 Plant diversity in tropical forests: a review of mechanisms of species coexistence.—Oecologia 130: 1–14. 28547014 10.1007/s004420100809

[9] Comita LS, Muller-Landau HC, Aguilar S, Hubbell SP. 2010 Asymmetric density dependence shapes species abundances in a tropical tree community.—Science 329: 330–332. 10.1126/science.1190772 20576853

[10] Mangan SA, Schnitzer SA, Herre EA, Mack KML, Valencia MC, Sanchez EI, et al 2010 Negative plant-soil feedback predicts tree-species relative abundance in a tropical forest.—Nature 466: 752–755. 10.1038/nature09273 20581819

[11] John R, Dalling JW, Harms KE, Yavitt JB, Stallard RF, Mirabello M, et al 2007 Soil nutrients influence spatial distributions of tropical tree species.—Proc. Natl. Acad. Sci. 104: 864–869. 17215353 10.1073/pnas.0604666104 PMC1783405

[12] Law R, Dieckmann U. 2000 A Dynamical System for Neighborhoods Implant Communities.—Ecology 81: 2137–2148.

[13] Pacala SW, Tilman D. 1994 Limiting similarity in mechanistic and spatial models of plant competition in heterogeneous environments.—Am. Nat. 143: 222–257.

[14] Peters HA. 2003 Neighbour-regulated mortality: The influence of positive and negative density dependence on tree populations in species-rich tropical forests.—Ecol. Lett. 6: 757–765.

[15] Bertness MD, Callaway R. 1994 Positive Interactions in communities.—Trends Ecol. Evol. 9: 191–193. 10.1016/0169-5347(94)90088-4 21236818

[16] Brooker RW, Maestre FT, Callaway RM, Lortie CL, Cavieres LA, Kunstler G, et al 2008 Facilitation in plant communities: the past, the present, and the future.—J. Ecol. 96: 18–34.

[17] Uriarte M, Swenson NG, Chazdon RL, Comita LS, John Kress W, Erickson D, et al 2010 Trait similarity, shared ancestry and the structure of neighbourhood interactions in a subtropical wet forest: Implications for community assembly.—Ecol. Lett. 13: 1503–1514. 10.1111/j.1461-0248.2010.01541.x 21054732

[18] Valiente-Banuet A, Verdú M. 2008 Temporal shifts from facilitation to competition occur between closely related taxa.—J. Ecol. 96: 489–494.

[19] Maestre FT, Callaway RM, Valladares F, Lortie CJ. 2009 Refining the stress-gradient hypothesis for competition and facilitation in plant communities.—J. Ecol. 97: 199–205.

[20] Kitajima K, Mulkey SS, Wright SJ. 2005 Variation in crown light utilization characteristics among tropical canopy trees.—Ann. Bot. 95: 535–547. 15585541 10.1093/aob/mci051 PMC4246798

[21] Uriarte M, Clark JS, Zimmerman JK, Comita LS, Forero-Montaña J, Thompson J. 2012 Multidimensional trade-offs in species responses to disturbance: implications for diversity in a subtropical forest.—Ecology 93: 191–205. 22486099 10.1890/10-2422.1

[22] Callaway RM, Walker LR. 1997 Competition and facilitation: a synthetic approach to interactions in plant communities.—Ecology 78: 1958–1965.

[23] Comita LS, Condit R, Hubbell SP. 2007 Developmental changes in habitat associations of tropical trees.—J. Ecol. 95: 482–492.

[24] Jordano P, Bascompte J, Olesen JM. 2003 Invariant properties in coevolutionary networks of plant–animal interactions.—Ecol. Lett. 6: 69–81.

[25] Pacala SW, Rees M. 1998 Models suggesting field experiments to test two hypotheses explaining successional diversity.—Am. Nat. 152: 729–737. 10.1086/286203 18811347

[26] Bruijnzeel LA, Scatena FN, Hamilton L. 2011 Tropical Montane Cloud Forest Science for Conservation and Management. Cambridge University Press.—Cambridge University Press.

[27] Condit R. 1998 Tropical forest census plots Springer Science & Business Media.

[28] Hubbell SP. 1999 Light-Gap Disturbances, Recruitment Limitation, and Tree Diversity in a Neotropical Forest.—Science 283: 554–557. 9915706 10.1126/science.283.5401.554

[29] De Cáceres M, Legendre P, Moretti M. 2010 Improving indicator species analysis by combining groups of sites.—Oikos 119: 1674–1684.

[30] Ripley BD. 1979 Tests of randomness’ for spatial point patterns.—J. R. Stat. Soc.—Ser. B Stat. Methodol.: 368–374.

[31] Illian J, Penttinen A, Stoyan H, Stoyan D. 2008 Statistical Analysis and Modelling of Spatial Point Patterns.—Wiley.

[32] Dale MRT. 1999. Spatial pattern analysis in plant ecology.—Cambridge, United Kingdom

[33] Goreaud F, Pélissier R. 2003 Avoiding misinterpretation of biotic interactions with the intertype K 12—function : population independence vs. random labelling hypotheses.—J. Veg. Sci. 14: 681–692.

[34] Ledo A, Condés S, Montes F. 2011 Intertype mark correlation function: A new tool for the analysis of species interactions.—Ecol. Modell. 222: 580–587.

[35] Clark DA, Clark DB. 1992 Life history diversity of canopy and emergent trees in a neotropical rain forest.—Ecological monographs 62: 315–344.

[36] Pardos M, Montes F, Aranda I, Cañellas I. 2007 Influence of environmental conditions on germinant survival and diversity of Scots pine (*Pinus sylvestris* L.) in central Spain.—Eur. J. For. Res. 126: 37–47.

[37] Loosmore NB, Ford ED. 2006 Statistical inference using the G or K point pattern spatial statistics.—Ecology 87: 1925–1931. 16937629 10.1890/0012-9658(2006)87[1925:siutgo]2.0.co;2

[38] Montgomery R, Chazdon R. 2002 Light gradient partitioning by tropical tree seedlings in the absence of canopy gaps.—Oecologia 131: 165–174. 28547683 10.1007/s00442-002-0872-1

[39] Hardin G. 1960 The competitive exclusion principle.—Science 131: 1292–1297. 14399717 10.1126/science.131.3409.1292

[40] Bagchi R, Henrys PA, Brown PE, Burslem DFRP, Diggle PJ, Gunatilleke CVS, et al 2011 Spatial patterns reveal negative density dependence and habitat associations in tropical trees.—Ecology 92: 1723–1729. 21939068 10.1890/11-0335.1

[41] Sheil D, Burslem DFRP. 2003 Disturbing hypotheses in tropical forests.—Trends Ecol. Evol. 18: 18–26.

