# Supplementary material for: Nature and Age of Neighbours Matter: Interspecific Associations among Tree Species Exist and Vary across Life Stages in Tropical Forests
Source: PLoS One. 2015 Nov 18;10(11):e0141387. doi: 10.1371/journal.pone.0141387 (PMC4651535; doi:10.1371/journal.pone.0141387)
Supplement: S1 Table — Ms refers to Morphospecies. (DOCX) [file pone.0141387.s002.docx]

| **Family** | **Specie** | **Shade tolerance** | **Life-form** | **N** |
| --- | --- | --- | --- | --- |
| Acanthaceae | *Aphelandra canthifolia Hook.* | Medium-tolerant | Mid-story | 55 |
| Actinidiaceae | *Saurauria* Ms | Gap | Under-story | 6 |
| Araliaceae | *Oreopanax Raimondii* Harms*.* | Shade-tolerant | Emergent | 50 |
| Bignonaceae | *Delostoma integrifolium* D. Don | Shade-tolerant | Canopy | 71 |
| Boraginaceae | *Tournefortia* Ms2 | Medium-tolerant | Under-story | 38 |
| Compositae | *Critoniopsis sevillana* (Cuatrec.) H.Rob. | Shade-tolerant | Canopy | 94 |
| Compositae | *Fulcaldea laurifolia* (Humboldt andBonpland) Poiret ex Lessing | Shade-tolerant? | Mid-story | 4 |
| Compositae | *Senecio* Ms | Gap | Mid-story | 48 |
| Elaeocarpaceae | *Vallea stipularis* Mutis ex L.f. | Shade-tolerant | Mid-story | 1 |
| Guttiferae | *Clusia flaviflora*Engl | Shade-tolerant | Canopy | 1 |
| Icacinaceae | *Cironella incarum* (J.F.Macbr.) R.A.Howard | Shade-tolerant | Canopy | 22 |
| Icacinaceae | *Citronella* Ms | Shade-tolerant | Under-story | 1 |
| Lauraceae | Nectandra Ms | Shade-tolerant | Mid-story | 2 |
| Lauraceae | Ocotea Ms1 | Shade-tolerant | Canopy | 27 |
| Lauraceae | Ocotea Ms2 | Shade-tolerant | Mid-story | 5 |
| Lauraceae | *Persea* Ms | Shade-tolerant | Emergent | 84 |
| Melastomataceae | *Miconia media* (D. Don) Naudin | Gap | Under-story | 74 |
| Melastomataceae | *Miconia denticulata* Naudin | Gap | Under-story | 78 |
| Melastomataceae | *Miconia firma* Macbr. | Gap | Under-story | 219 |
| Meliaceae | *Guarea* Ms | Shade-tolerant | Canopy | 5 |
| Meliaceae | *Ruagea glabra* Triana and Planchon | Shade-tolerant | Canopy-emergent | 143 |
| Monimiaceae | *Siparuna muricata* (Ruiz and Pavón) A.DC. | Shade-tolerant | Mid-story-canopy | 37 |
| Moraceae | *Morus insignis*Bureau*.* | Shade-tolerant | Canopy | 62 |
| Myrsinaceae | *Myrsine latifolia* (Ruiz andPavon) Sprengel | Gapto Medium | Mid-story | 15 |
| Myrsinaceae | Parathesis Ms | Shade-tolerant | Under-story | 341 |
| Myrtaceae | *Eugenia* Ms | Shade -tolerant | Under-story | 324 |
| Myrtaceae | *Myrcianthes fimbriata* (Kunth) McVaugh | Medium-tolerant | Mid-story | 23 |
| Myrtaceae | *Myrcianthes discolor* (Kunth) McVaugh | Medium-tolerant | Mid-story | 12 |
| Myrtaceae | *Myrcianthes* Ms | Medium-tolerant | Mid-story | 21 |
| Papaveraceae | *Bocconia integrifolia* Humb. andBonpl. | Medium-tolerant | Under-story | 1 |
| Piperaceae | *Piper elongatum* (Poir. ex Vahl) C.DC | Gap | Under-story | 739 |
| Polygalaceae | *Monnina pilosa* H. B. and K. var. g*labrescens*Ferreyra | Gap | Under-story | 3 |
| Polygalaceae | *Monnina ligustrifolia* Kunth in Humboldt and al | Medium-tolerant | Under-story | 4 |
| Ranunculaceae | *Clematis* Ms | Medium-tolerant | Under-story | 6 |
| Rubiaceae | *Palicourea* Ms | Shade-tolerant | Mid-story | 1 |
| Rubiaceae | *Randia boliviana* Rusby | Shade-tolerant | Under-story | 1 |
| Sabiaceae | *Meliosma* Ms1 | Shade-tolerant | Emergent | 26 |
| Sabiaceae | *Meliosma* Ms2 | Shade-tolerant | Emergent | 86 |
| Saxifragaceae | *Escallonia* Ms | Shade-tolerant | Emergent | 3 |
| Solanaceae | *Cestrum auriculatum* L'Hér | Shade-tolerant | Mid-story | 67 |
| Solanaceae | *Iochroma squamosum* Leiva andQuipuscoa | Medium-tolerant | Mid -story | 63 |
| Solanaceae | *Lycianthes inaequilatera (Rusby) Bitter.* | Medium-tolerant | Under-story | 144 |
| Solanaceae | *Solanum* Ms1 | Gap | Under-story | 1307 |
| Solanaceae | *Solanum* Ms2 | Medium-tolerant | Mid-story | 109 |
| Solanaceae | *Solanum oblongifolium* Dunal | Shade-tolerant | Under-story | 2 |
| Urticaceae | *Boehmeria caudata* Sw. | Shade-tolerant | Under-story | 2 |
| Winteraceae | *Drimys* Ms | Shade-tolerant | Emergent-canopy | 70 |
| -- | Morphospecie 1 | -- | Under-story | 1 |
| -- | Morphospecie 2 | -- | Under-story | 1 |
| -- | Morphospecie 3 | -- | Under-story | 3 |
| -- | Morphospecie 4 | -- | Under-story | 1 |
| -- | Morphospecie 5 | -- | Under-story | 2 |
| -- | Morphospecie 6 | -- | Under-story | 2 |
