# Supplementary material for: Nature and Age of Neighbours Matter: Interspecific Associations among Tree Species Exist and Vary across Life Stages in Tropical Forests
Source: PLoS One. 2015 Nov 18;10(11):e0141387. doi: 10.1371/journal.pone.0141387 (PMC4651535; doi:10.1371/journal.pone.0141387)
Supplement: S2 Table — (DOCX) [file pone.0141387.s003.docx]

| **Habitat** | **Family** | **Species** | **Shade tolerance** | **Growth form** | **N** |
| --- | --- | --- | --- | --- | --- |
| High plateau | Capparaceae | *Capparis frondosa* | -- | Shrubs | 2749 |
| High plateau | Myrtaceae | *Eugenia nesiotica* | Shade | Midstorey | 482 |
| High plateau | Sapotaceae | *Chrysophyllum cainito* | Gap | Canopy | 147 |
| Low plateau | Boraginaceae | *Cordia bicolor* | Gap | Midstorey | 658 |
| Low plateau | Burseraceae | *Protium panamense* | Shade | Midstorey | 2853 |
| Low plateau | Burseraceae | *Tetragastris panamensis* | Shade | Canopy | 4493 |
| Low plateau | Melastomataceae | *Mouriri myrtilloides* | -- | Shrubs | 6540 |
| Low plateau | Ochnaceae | *Ouratea lucens* | -- | Shrubs | 1227 |
| Low plateau | Rubiaceae | *Coussarea curvigemmia* | Shade | Understorey | 2058 |
| Low plateau | Simaroubaceae | *Simarouba amara* | Intermediate | Canopy | 1477 |
| Slope | Annonaceae | *Unonopsis pittieri* | Shade | Midstorey | 621 |
| Slope | Annonaceae | *Xylopia macrantha* | Shade | Midstorey | 1414 |
| Slope | Chrysobalanaceae | *Hirtella triandra* | Shade | Midstorey | 4566 |
| Slope | Euphorbiaceae | *Drypetes standleyi* | Shade | Canopy | 2180 |
| Slope | Lauraceae | *Ocotea oblonga* | Intermediate | Canopy | 162 |
| Slope | Lauraceae | *Ocotea whitei* | Shade | Canopy | 374 |
| Slope | Moraceae | *Trophis caucana* | Shade | Understorey | 149 |
| Slope | Sapotaceae | *Chrysophyllum argenteum* | Gap | Canopy | 670 |
| Stream | Euphorbiaceae | *Croton billbergianus* | Gap | Understorey | 468 |
| Stream | Moraceae | *Brosimum alicastrum* | Intermediate | Canopy | 892 |
| Stream | Moraceae | *Trophis caucana* | Shade | Understorey | 149 |
| Swamp | Apocynaceae | *Tabernaemontana arborea* | Intermediate | Canopy | 1593 |
| Swamp | Rhizophoraceae | *Cassipourea elliptica* | Shade | Midstorey | 1069 |
| Swamp | Rubiaceae | *Alibertia edulis* | Shade | Understorey | 370 |
| Swamp | Rubiaceae | *Psychotria graciliflora* | -- | Shrubs | 53 |
